# Galvanic vestibular stimulation to rehabilitate postural instability in Parkinson's disease

**DOI:** 10.1055/s-0045-1806812

**Published:** 2025-04-22

**Authors:** Anna Paula Batista de Ávila Pires, Ludimila Labanca, Paulo Pereira Christo, Maurício Campelo Tavares, Jordana Carvalhais Barroso, Maria Luiza Diniz, Denise Utsch Gonçalves

**Affiliations:** 1 Universidade Federal de Minas Gerais, Escola de Medicina, Departamento de Oftalmologia e Otorrinolaringologia, Belo Horizonte MG, Brazil.; 2 Universidade Federal de Minas Gerais, Escola de Medicina, Programa de Pós-Graduação em Ciências Fonoaudiológicas, Belo Horizonte MG, Brazil.; 3 Universidade Federal de Minas Gerais, Escola de Medicina, Departamento de Fonoaudiologia, Belo Horizonte MG, Brazil.; 4 Universidade Federal de Minas Gerais, Escola de Medicina, Departamento de Clínica Médica, Belo Horizonte MG, Brazil.; 5 Instituto de Ensino e Pesquisa Santa Casa de Belo Horizonte, Belo Horizonte MG, Brazil.; 6 Universidade Federal de Santa Catarina, Laboratório de Pesquisas em Processamento Digital de Sinais, Florianópolis SC, Brazil.

**Keywords:** Parkinson Disease, Neurodegenerative Diseases, Postural Balance, Electric Stimulation, Rehabilitation

## Abstract

**Background** Galvanic vestibular stimulation (GVS) is a non-invasive technique employed to rehabilitate balance by delivering low-intensity, short-duration electrical stimulation to the mastoid bones, effectively activating the vestibulospinal tract.

**Objective** To evaluate the effects of GVS on balance in patients with Parkinson's disease (PD) and postural instability.

**Methods** In this clinical study, 25 PD patients with postural instability in the ON phase (best effect of dopaminergic medication) underwent GVS. Balance was assessed using the Berg Balance Scale (BBS), the Timed Up and Go (TUG) test, and posturography on a force platform. Electrical current intensity was progressively increased between the mastoids, starting at 1.0 mA and reaching 3.5 mA by the 6th session, with this level maintained until the 8th session. Stimulation duration began at 9 minutes in the 1st session, increased to 30 minutes by the 3rd session, and was sustained through the 8th session.

**Results** A blinded comparison of pre- and post-GVS evaluations demonstrated significant improvements in BBS ( *p* = 0.00001) and TUG ( *p* = 0.00003) scores. Posturography showed an increase in the stability limit area ( *p* = 0.026) and the general balance index ( *p* = 0.001).

**Conclusion** In the therapeutic management of postural instability in PD, GVS emerges as a promising complementary strategy for enhancing balance. Further research is needed to determine whether these improvements persist after GVS cessation.

**Registration of Clinical Trial:** https://ensaiosclinicos.gov.br/rg/RBR-22j8728 .

## INTRODUCTION

Postural instability is a late-onset clinical manifestation of Parkinson's disease (PD), which shows limited improvement with pharmacological treatment and is a major contributor to falls in PD patients. 1 Vestibular dysfunction in PD is associated with impairments in the connection between the basal ganglia and the vestibulo-thalamo-striatal pathway. 2 Additionally, the pedunculopontine nucleus, which is also affected in PD, plays a key role in the modulation of postural control and balance. 3 4 5 Therefore, central vestibular system dysfunction and its impact on efferent vestibular reflexes are integral components of the pathophysiology of postural instability in PD.

Galvanic vestibular stimulation (GVS) is a non-invasive technique that stimulates the vestibular system, including sensory organs, neural pathways, vestibular nuclei, and cortical areas receiving integrated vestibular inputs. It modulates neural discharge by influencing the spike encoder in axon terminals, generating controlled vestibular signals that are unaffected by somatosensory inputs and are perceived by the nervous system as constant angular acceleration. 6

Galvanic vestibular stimulation can be delivered in two modes: suprathreshold stimulation, in which the subject perceives stimulation as a sensation of body oscillation, and subthreshold stimulation, in which the stimulation is below the cutaneous threshold, and no perception of sway occurs. 6 7 8 9 A specific subthreshold variation known as noisy GVS applies stochastic resonance to the peripheral vestibular system via a band-limited noisy current, improving balance in conditions such as bilateral vestibulopathy and PD. 7 8 10

The suprathreshold vestibular stimulation consists of direct current GVS that mimics constant head motion signals directed toward the cathode (positive pole) and elicits compensatory postural and oculomotor responses toward the anode (negative pole). 11 Modifying the positions of the cathode and anode strongly influences postural adjustments and balance. 12 The semicircular canals, otolithic organs, and associated vestibular nerves are activated during GVS, leading to modulation of posture, spatial orientation, oculomotor response and balance. 13

The vestibular afferent system comprises irregular and regular firing axons, reflecting their diverse properties. Irregular firing axons, which form a significant proportion of primary neurons, are highly sensitive to sensory inputs, efferent activation, and galvanic currents, making them more responsive to noisy GVS and recruited at lower current amplitudes compared with the regular firing fibers. 9 14 Conversely, regular firing axons, predominant in secondary vestibular neurons, exhibit higher depolarization thresholds and reduced responses to low-intensity currents. 14 15 Notably, irregular axons adapt to firing-rate changes during noisy GVS, while regular axons do not show such adaptation under prolonged suprathreshold stimulation. 9

Galvanic vestibular stimulation has been applied to enhance vestibular function in patients with bilateral vestibulopathies, 16 uncompensated unilateral vestibulopathies 17 and to improve balance in PD. 18 19 20 21 22 23 It was shown to improve anxiety, 24 cognition, 25 and memory. 26 The use of GVS in clinical practice has been advancing as a result of its favorable characteristics, such as objectivity, safety, low cost, easy-to-learn technique, and minimal discomfort to the patient. 27

Previous studies have explored noisy GVS in PD-related postural instability, with varying results. 18 20 21 28 29 Pal et al. 20 demonstrated improved posturography parameters in PD patients using GVS at 0.1, 0.3, and 0.5 mA, although sample size was a limitation. Similarly, Okada et al. 18 reported reduced anterior trunk flexion angles after GVS, especially with eyes closed. Samoudi et al. 28 highlighted that GVS improved postural control during peak and off-medication states, indicating it effectively reduced levodopa-related fluctuations. Kataoka et al. 21 and Tran et al. 29 further confirmed the positive effects of GVS on postural parameters in PD patients, though limitations included small sample sizes and methodological constraints. Despite these promising findings, existing evidence remains limited due to low statistical power, heterogeneous stimulation protocols, and methodological weaknesses. 8

In the current study, we propose the application of GVS in stepwise current intensities using higher-amplitude pulses (> 0.9 mA) to enhance postural stability in PD patients. Our hypothesis is that increasing current intensities will effectively stimulate secondary polysynaptic neurons and potentially influence the central vestibular circuits, thereby improving postural balance in PD.

## METHODS

The present study was approved by the Human Research Ethics Committee of the participating institutions, and all participants provided written informed consent prior to enrollment. The procedures adhered to the ethical standards outlined by the institutional review board and complied with the principles of the Declaration of Helsinki. The clinical trial was registered with the Brazilian Clinical Trials Registry under the identifier RBR-22j8728 ( https://ensaiosclinicos.gov.br/rg/RBR-22j8728 ).

### Subjects

The participants included 25 patients with PD and postural instability, followed at a PD reference center. The mean age was 68(± 10) years, and there were 17 male participants (68%). The mean time since postural instability onset was 9(± 3) years, ranging from 5 to 16 years ( Table 1 ). All patients were on regular levodopa therapy. Every participant completed the stimulation protocol and was evaluated using the tests before and after the intervention.

The PD dopaminergic medications were kept unchanged for 30 days prior to study inclusion. Postural instability was defined as two or more falls in the previous year and a score of 3 (stands safely but lacks postural response and falls without support) on item 12 of the Movement Disorder Society - Unified Parkinson's Disease Rating Scale (MDS-UPDRS) part III. 30 This test was conducted as described in the referenced study. 31 The exclusion criteria were:

- recurrent vertigo episodes, a single vertigo episode lasting over 30 minutes, or a history of vestibular disease;
- history of myelitis or stroke;
- immobility syndrome (e.g., requiring a wheelchair, walking aids, or presenting with gait freezing);
- use of a pacemaker or other implanted electronic devices affected by GVS;
- orthopedic or neurological comorbidities affecting balance;
- use of drugs that suppress vestibular function (e.g., benzodiazepines, dimenhydrinate, meclizine, flunarizine, and cinnarizine).

The tests and GVS were conducted during the ON phase of dopaminergic medication. No patient had dyskinesia, which can interfere with the pre- and poststimulation assessments.

### Procedure

Participants underwent an 8-week protocol of GVS applied in incremental current steps, with the intervention conducted in a blinded before-and-after comparison design. The primary outcomes were improvements in body balance, evaluated through posturographic parameters, and functionality, assessed using the Timed Up and Go (TUG) test and the Berg Balance Scale (BBS). The cutoff for the TUG test was time equal or greater than 15 seconds, previously associated with reduced functionality in PD patients. 32 The cutoff for the BBS was a score lower than or equal to 49 points, linked to decreased performance in daily activities and increased fall risk. 33

Assessments were performed one week before and one week after the GVS protocol. The order of tests was TUG, BBS, and balance evaluation using a force platform. Two examiners conducted the tests—one managing execution and the other recording measurements. Data entry was blinded to patient identity and whether results corresponded to pre- or poststimulation. Database integrity was verified twice.

Posturography was performed using a force platform (HORUS - Contronic, Florianópolis, SC, Brazil), evaluating visual, somatosensory, and vestibular contributions to balance. Patients stood on the platform in the anteroposterior (AP) and mediolateral (ML) planes. Stability limits were tested by asking participants to lean as far as possible using ankle strategies without trunk movements or stepping. Then, they underwent sensory organization tests on the platform in the following conditions:

- C1) eyes open with a fixed target on a stable surface;
- C2) eyes closed on a stable surface;
- C3) eyes open with visual conflict on a stable surface (optokinetic training with words);
- C4) eyes open with a fixed target on an unstable surface (cushion);
- C5) eyes closed on an unstable surface; and
- C6) eyes open with visual conflict on an unstable surface (read words on a moving image panel). 34

For each of the six conditions, the individual's displacement was evaluated, and the score was obtained in percentage (%). Eyes closed on an unstable surface (C5) is the condition that best evaluates the contribution of the vestibular system to maintaining postural balance. The quantification of the results ranged from 100% (no displacement recorded by the platform's sensors) to 0% (fall in any direction). Afterward, the equipment software calculated the somatosensory balance index ([C2%/C1%]*100), visual balance index [C4%/C1%]*100), vestibular visual balance index ([%C5/%C1]*100) and general balance index ([C1% + C2% + C3% + C4% + C5% + C6%/6]*100). 35 The sensory organization tests provide quantitative information on visual, proprioceptive and labyrinthine representation in the control of body balance. 36 The calculation of the general balance index informs about the participation of the visual, vestibular, and somatosensory systems, not giving priority to any of them. All data were automatically entered in the equipment software. Regarding the posturographic parameters, the confidence ellipse area refers to the area that encompasses 95% of the points of an individual's center of pressure on the force platform during the tests, and the smaller the confidence ellipse, the smaller the individual's body displacement, demonstrating greater body stability after GVS. The path length refers to the total length of the trajectory of the individual's center of pressure during the tests, and the total mean velocity refers to the length of the trajectory of the center of pressure through the force platform divided by the time of measurement. The lower the path length and total mean velocity, the greater the individual's stability during posturographic measures.

Body movements in each condition of stimulation were measured at intervals of at least 30 seconds, at most 1 minute, and preferably 45 seconds. The following parameters were measured: stability limit area (SLA); 95% confidence ellipse (CE); path length (PL); and total mean velocity (TMV). Stability limit area is the maximum limit of body displacement in the anteroposterior and mediolateral directions using the ankle strategy and without moving the center of pressure. Confidence ellipse is the area that encompasses 95% of the points of the person's center of pressure on the force platform during the tests. The lower the CE, the smaller the person's body displacement, indicating better postural stability. Path length is the total length of the path over the platform taking as reference the person's center of pressure during the tests. Total mean velocity is the PL divided by the measure of time. The lower the PL, the better the person's stability during the tests.

### Stimulation

**Figure 1:**
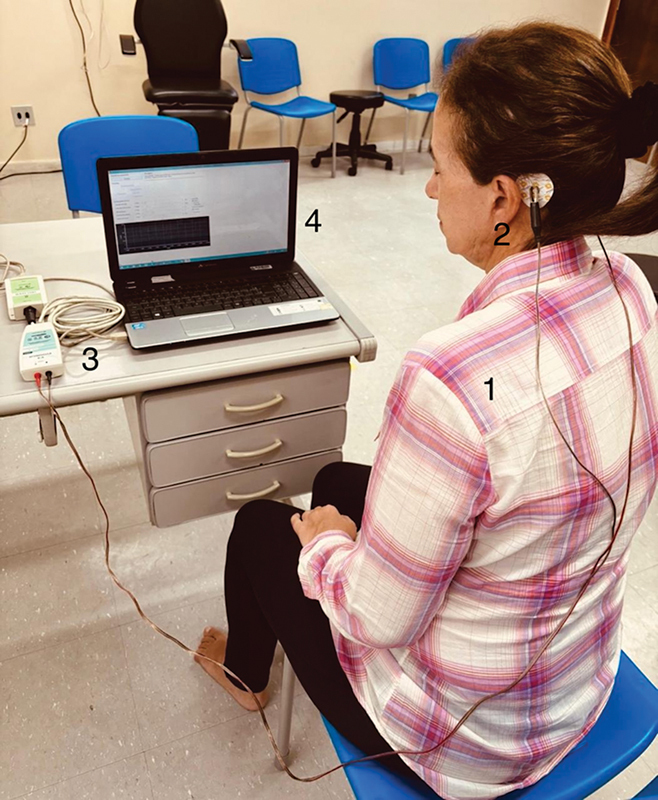
Galvanic vestibular stimulation: 1) the individual remained seated, barefoot, with eyes closed, wearing no object that might conduct electricity; 2) stimulation electrodes; 3) GVS-generating equipment; 4) software to control the time and intensity of stimulation.

Participants underwent weekly GVS sessions over eight weeks. Each session consisted of 3 series of stimulations, with a 1-minute rest between series. The intervention began with the lowest current, shorter stimulation time, and fewest repetitions, which was gradually increased throughout the treatment, according to the patient's tolerance. During GVS, patients were seated, shoeless, with their eyes closed and without any objects that could be good electrical conductors ( Figure 1 ). The sessions were supervised by an audiologist specializing in GVS.

The stimulation protocol used in the current study is described in Table 2 and consisted of one session of stimulation per week during eight consecutive weeks. Each weekly session consisted of three series of stimulation. The rest interval after each series of stimulation was 1 minute.

**Table 1:** Comparison of 25 patients with Parkinson's disease regarding sex, age, and disease duration

Gender (%)	Mean age (years)	*p* -value*	Mean disease duration (years)	*p* -value*
Females 8 (32)	70.5 ± 9.7	0.49	8.6 ± 2.1	0.56
Males 17 (68)	67.6 ± 9.4		8.9 ± 3.1	

Note: *Student's *t* -test.

**Table 2:** Galvanic vestibular Stimulation (GVS) in 8 sessions, 1 session per week, 3 series per session, totaling 8 weeks of stimulation

GVS	Session							
	1st	2nd	3rd	4th	5th	6th	7th	8th
**Series**	[1.0/1/3]	[2.0/2/3]	[2.0/2/5]	[2.5/2/5]	[2.5/2/5]	[2.5/2/5]	[3.0/2/5]	[3.0/2/5]
	[1.5/1/3]	[2.5/2/3]	[2.5/2/5]	[3.0/2/5]	[3.0/2/5]	[3.0/2/5]	[3.0/2/5]	[3.5/2/5]
	[2.0/1/3]	[2.5/2/3]	[2.5/2/5]	[3.0/2/5]	[3.0/2/5]	[3.5/2/5]	[3.5/2/5]	[3.5/2/5]

Notes: [Current in mA/stimulus duration in minutes/number of stimulus repetitions]. The intensity of the stimulation current, and/or the duration of the stimulus, and/or the number of repetitions were progressively increased within the same session, according to the patient's tolerance.

The equipment (Contronic) provided an electrical current in a binaural and bipolar configuration generated by a constant current stimulator with a single rectangular stimulus and alternating polarity. The disposable circular surface electrodes measuring 3 cm in diameter (Valutrode – model CF3200–Axelgaard Manufacturing Co., Ltd., Fallbrook, CA, USA) were fixed on both mastoid processes. The current was gradually increased with a ramp-up period of 5s. The current intensity was increased by 1.0 mA until reaching 3.5 mA in the 6 ^th^ week and then maintained until the 8 ^th^ week. The stimulation time was gradually increased from 1 to 2 minutes, and the number of consecutive stimulus repetitions was 3 until the 2 ^nd^ week and then increased to 5, which was maintained until the 8 ^th^ week. Therefore, the stimulation time per series was 9 minutes in the 1 ^st^ session and increased to 30 minutes in the 3 ^rd^ session and then this duration was maintained until the 8 ^th^ session.

### Statistics

Statistical analysis was performed using the IBM SPSS Statistics for Windows (IBM Corp., Armonk, NY, USA), version 23. Variables with symmetrical distribution (e.g., age) were expressed as mean ± standard deviation (SD) values. Asymmetrical variables were presented as medians with interquartile ranges. Categorical data were expressed as frequencies. The Wilcoxon test was used to compare pre- and post-GVS variables, with significance set at *p* < 0.05.

## RESULTS

All participants successfully completed the GVS protocol. Patients described experiencing a tolerable tingling or mild shock sensation at the stimulation site, with no additional side effects reported. Based on medical records, the time since the onset of postural instability ranged from 5 to 16 years, with a median of 9 years (interquartile range: 7–10 years).

**Figure 2:**
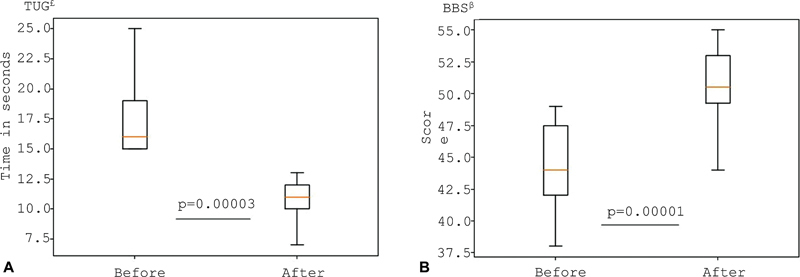
Notes: ^£^ Time in seconds. ^β^ Higher scores indicate a perception of better balance. Wilcoxon's test. Comparative analysis of the Timed Up and Go Test (TUG) ^£^ and Berg Balance Scale (BBS) ^β^ before and after eight sessions of galvanic vestibular stimulation.

The results of the BBS and TUG tests before and after the intervention are shown in Figure 2 .

**Figure 3:**
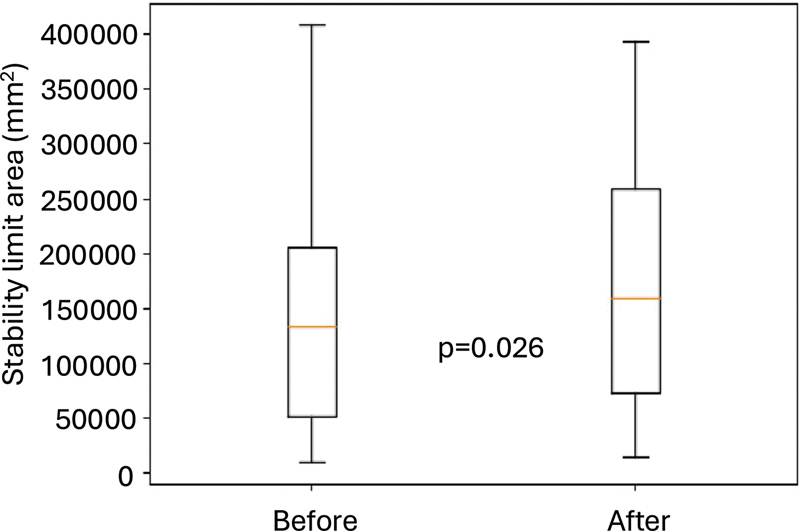
Note: Wilcoxon's test. Comparison of stability limit area (mm ^2^ ) before and after eight sessions of galvanic vestibular stimulation.

Both BBS scores and TUG test times significantly improved following GVS. Results from the stability limit assessment conducted on a force platform are presented in Figure 3 . The increase in the SLA after GVS indicates improved postural balance.

The visual, vestibular, somatosensory, and general balance indices are summarized in Table 3 . Improvements were noted in the overall balance, vestibular, and visual indices following GVS.

**Table 3:** Posturographic parameters of the visual, vestibular, somatosensory indexes as well as the overall balance index before and after eight sessions of galvanic vestibular stimulation

Posturographic parameters	Galvanic vestibular stimulation		*p* -value
	Before	After	
Vestibular index	76 (49/87)	91 (84/97)	0.00002
Visual index	90 (79/93)	94 (88/97)	0.007
Somatosensory index	100 (95/103)	99 (97/100)	0.276
Balance index	84 (59/91)	94 (85/95)	0.001

Notes: Values expressed as median (first/third quartiles). Wilcoxon's test.

Changes in the SLA, CE, PL, TMV, and posturography scores under each condition are detailed in Table 4 . Specifically, in C5 (eyes closed on an unstable surface), there was a notable reduction in the CE area, PL, and TMV, alongside an increase in the balance score, reflecting better postural stability after GVS.

**Table 4:** Postural parameters of the stability limit area, confidence ellipse, path length, total mean velocity, and score for each condition in posturography before and after eight sessions of galvanic vestibular stimulation applied to 25 individuals with Parkinson's disease and instability

Condition	Parameter	Galvanic vestibular stimulation		*p* -value
		Before	After	
C1 **–** eyes open with a fixed target on a stable surface	Confidence ellipse area	849 (384/2,041)	424 (237/1,143)	0.313
	Path length	626 (436/1,502)	549 (358/902)	0.276
	Mean velocity	16 (12/36)	15 (9/24)	0.518
	Score	94 (87/97)	97 (94/99)	0.032
C2 **–** eyes closed on a stable surface	Confidence ellipse area	773 (469/1,798)	671 (262/1,157)	0.677
	Path length	738 (537/1,518)	689 (474/1,021)	0.242
	Mean velocity	21 (14/43)	22 (14/32)	0.667
	Score	91 (86/97)	97 (91/99)	0.135
C3 **–** eyes open with visual conflict on a stable surface	Confidence ellipse area	782 (489/3,552)	578 (274/1,361)	0.083
	Path length	730 (498/2,378)	597 (440/1,320)	0.221
	Mean velocity	21 (12/70)	15 (12/33)	0.493
	Score	91 (71/96)	97 (91/99)	0.013
C4 **–** eyes open with a fixed target on an unstable surface	Confidence ellipse area	2,090 (1,471/4,008)	1,334 (1,171/2,047)	0.021
	Path length	1,039 (783/1,539)	782 (688/968)	0.098
	Mean velocity	28 (22/46)	25 (18/30)	0.346
	Score	81 (65/90)	93 (83/85)	0.002
C5 **–** eyes closed on an unstable surface	Confidence ellipse area	4,378 (2860/5,963)	2,370 (1517/3,113)	< 0.001
	Path length	1,570 (1,190/2,433)	1,110 (970/1,513)	0.006
	Mean velocity	55 (38/85)	35 (28/48)	0.002
	Score	68 (31/84)	89 (76/93)	< 0.001
C6 **–** eyes open with visual conflict on an unstable surface	Confidence ellipse area	2,903 (1,613/4,623)	1,760 (1,253/2,620)	0.042
	Path length	1,421 (883/2,315)	961 (695/1,302)	0.048
	Mean velocity	35 (25/75)	25 (21/39)	0.088
	Score	76 (49/90)	91 (80/95)	0.002

Notes: Values expressed as median (first/third quartiles). Wilcoxon's test.

The full dataset is available as **Supplementary Material** available at https://www.arquivosdeneuropsiquiatria.org/wp-content/uploads/2025/01/ANP-2024.0200-Supplementary-Material.xlsx

## DISCUSSION

In the current study, postural instability was observed at a median of 9 years following PD onset, consistent with it being a late-stage manifestation. Improvements in BBS scores suggest that participants experienced greater ease and confidence in performing daily activities postintervention. Similarly, TUG times decreased after GVS ( Figure 2 ). 32

Posturography demonstrated improvements in SLA post-GVS ( Figure 3 ) and overall balance ( Table 3 ). Condition 5 (eyes closed on an unstable surface) showed significant improvements in the balance index, emphasizing the role of GVS in stimulating vestibular function. 37 GVS primarily activates vestibular afferents, and its efferent pathways (e.g., the vestibulo-ocular reflex) likely contributed to the observed enhancement in the visual index ( *p* = 0.007, Table 3 ).

Galvanic vestibular stimulation can improve postural instability in PD through stimulation of cholinergic areas related to balance of the vestibulo-thalamic-striatal system. 3 One possible mechanism of the effects of GVS is the activation of the bilateral vestibulospinal tract and the activation of the antigravity muscles leading to an improvement of the anterior bending posture. 18 Therefore, the important contribution of the increased vestibular index to the overall balance after GVS suggests a vital role of the vestibular pathways in improving postural instability in PD ( Table 3 ).

The behavioral response observed during GVS applied in step current is the complex product of the galvanic stimulus that activates the central vestibular system followed by processing/weighting of different afferent signals. It is a safe, inexpensive, easily applied method with few transitory adverse effects related to the moment of stimulation. The main complaints are mild itching and tingling on the stimulation site, described by ∼ 10% of the patients, and pain underneath and around the stimulus electrodes. 27 In the present study, the participants did not report any discomfort even with the higher current. The use of large surface electrodes (∼1 cm ^2^ ) on the mastoid and generous covering with electrode gel help to minimize the risk of skin irritations, burns, and patient discomfort. 38 We increased the current gradually with a ramp-up period of 5s, which decreases discomfort. 37

The noise-induced facilitation of vestibulospinal reflexes via stochastic resonance has been studied to treat balance disorders such as bilateral vestibulopathy 7 and PD. 18 19 Taking into consideration the GVS-induced responses, we proposed another GVS waveform, a step current protocol. The lowest current intensities used in noise GVS, that is generally below 1.0 mA, appear to recruit hair cells, whereas larger currents used in the present study become more effective in directly modulating the afferent spike activity. Galvanic vestibular stimulation-evoked behavioral responses (oculomotor, postural, and perceptual) generated by larger current favors central convergence and integration of the signals. Therefore, our hypothesis is that higher current intensity is more effective to promote the activation of central vestibular circuits compared with the intensity of current used in noisy GVS.

Regarding the technical parameters of GVS for rehabilitation purposes, there are variable characteristics in relation to the site of stimulation (inner ear or mastoids) amplitude of the current, waveform (current in steps or pulses, sinusoidally modulated, band-limited noisy) whether unilateral or bilateral, time and frequency of stimulation, number of repetitions, and duration of the treatment. This variation is less observed for the stimulus site (mastoid) and the type of electrode (surface). It is noteworthy that the protocol used in the present study is just one among several options for GVS stimulation protocols. 26 33

The limitations of this study have to be considered. There was no sham group. Despite this limitation, postural balance improvement was observed based on posturography and TUG test results, which are objective tests. The analysis compared blindly the results before and after the intervention. Therefore, the evidence suggests that the improvement was caused by the intervention and not by a learning effect or a placebo response. The sample size consisted of 25 participants, and all the participants had a before and after comparison. A recent meta-analysis of GVS in PD included 5 studies whose sample sizes ranged from 5 to 13 PD participants. 8 The selection of our participants was not randomized. There may have been a selection bias toward acceptance by proactive individuals with fewer comorbidities, such as mood disorders. These facts may have induced to the selection of a sample of patients in better balance conditions. However, despite these biases, all participants reported instability, which improved after the intervention, according to the blind analysis. Given the importance of improving balance in PD patients who are still functional, the study results were quite favorable as subjectively, according to BBS, and objectively, according to posturographic measurements, the improvement of body balance occurred after the intervention. Further studies are necessary to confirm whether the gain in body balance is maintained after stopping GVS.
